# RdxA-independent mechanism of *Helicobacter pylori* metronidazole metabolism

**DOI:** 10.3389/fmicb.2025.1553734

**Published:** 2025-03-26

**Authors:** Yakun Zhao, Lihua He, Lu Sun, Wentao Liu, Hairui Wang, Jianzhong Zhang, Yanan Gong, Xiaohui Wang

**Affiliations:** ^1^National Key Laboratory of Intelligent Tracking and Forecasting for Infectious Diseases, National Institute for Communicable Disease Control and Prevention, Chinese Center for Disease Control and Prevention, Beijing, China; ^2^Department of Health Statistics, China Medical University, Shenyang, China; ^3^Department of Gastroenterology, The Sixth Medical Center, Chinese PLA General Hospital, Beijing, China

**Keywords:** *Helicobacter pylori*, metronidazole metabolism, RdxA-independent, NADH-quinone oxidoreductase, metronidazole resistance

## Abstract

**Introduction:**

Metronidazole (MNZ) is widely used to treat *Helicobacter pylori* infection worldwide. However, due to excessive and repeated use, resistance rates have exceeded 90% in some regions. The mechanisms of MNZ resistance have been extensively studied, and RdxA has been identified as the primary enzyme responsible for MNZ activation. Mutations in RdxA, particularly termination mutations, can lead to high-level MNZ resistance.

**Methods:**

We identified a strain, ICDC15003s, which harbored RdxA termination mutation but remained highly susceptible to MNZ. To explore this phenomenon, we conducted comparative genomic and transcriptomic analyses to define RdxA-independent mechanisms of MNZ metabolism.

**Results and discussion:**

We found missense mutations in genes such as *yfkO*, *acxB*, *alr1*, *glk*, and *cobB*. Additionally, the expression of multiple genes, including TonB-dependent receptor and mod, significantly changed in resistant strains. Notably, the sequences and expression levels of known nitroreductases like FrxA and FdxB remained unchanged after induction of MNZ resistance, suggesting they were not responsible for MNZ sensitivity in ICDC15003s. Instead, transcriptional alterations were observed in genes encoding NADH-quinone oxidoreductase subunit (M, J, H and K), suggesting a potential compensatory mechanism for the loss of RdxA activity. We proposed that NADH-quinone oxidoreductase might serve as an RdxA-independent mechanism for MNZ metabolism and resistance through regulation of its expression levels. This discovery could provide new strategies to address MNZ resistance and aid in developing nitroimidazole antibiotics.

## 1 Introduction

*Helicobacter pylori* (*H. pylori*) infection can cause chronic gastritis, peptic ulcer disease, gastric carcinoma, and mucosa-associated lymphoid tissue lymphoma (Parsonnet et al., 1991; Uemura et al., 2001). The standard therapy has failed to achieve a success eradication rate of 80% and increasing antibiotic resistance of *H. pylori* is the principal reason for eradication failure (Malfertheiner et al., 2017). Metronidazole (MNZ) is widely used to treat *H. pylori* infection worldwide, however, due to the excessive and repeated use, MNZ resistance rates have reached above 90% in some areas (Molina-Infante et al., 2012; Su et al., 2013). Although MNZ resistance can be partially overcome by increasing dose, frequency, and duration of MNZ usage (Fallone et al., 2016), more than 70% of resistant strains were high-level resistance to MNZ (minimal inhibitory concentration, MIC >256 μg/mL), and even the maximum safe dose of MNZ could not overcome the resistance (Gong et al., 2023).

MNZ is a prodrug that must be activated by reducing the nitro group and transformed into a DNA-damaging hydroxylamine intermediate that reacts with multiple cellular targets (Dingsdag and Hunter, 2018; Leitsch, 2017). In *H. pylori*, RdxA, an oxygen-insensitive NADPH nitroreductase, is the primary enzyme responsible for MNZ activation (Olekhnovich et al., 2009). Mechanisms of MNZ resistance have been extensively investigated, which may depend on the reduction or abolition of activity of the electron carriers and the intracellular redox potential (Francesco et al., 2011; Kaakoush et al., 2009). It is accepted that inactivation of RdxA was mainly associated with MNZ resistance (Goodwin et al., 1998; Hashemi et al., 2019; Marques et al., 2019). The high-level MNZ resistance was a polygenic trait, with the procession from susceptible to high-level MNZ resistant beginning with mutations in RdxA and resulting from multiple mutations (Kaakoush et al., 2009). Previous studies revealed that RdxA termination mutation can directly lead to the loss of enzyme activity and high-level resistance to MNZ (Solca et al., 2000; Tankovic et al., 2000; Mannion et al., 2021). Our previous study, through sequence analysis of 511 clinical strains, demonstrated a strong correlation between RdxA truncation mutations and MNZ resistance. Specifically, truncations occurring before the 70th amino acid position were associated with higher level of resistance (MIC >256 μg/mL) (Gong et al., 2023). Intriguingly, among these 511 strains analyzed, we identified a highly MNZ-susceptible strain ICDC15003s, that harbored a nonsense mutation in the *rdxA* gene, producing a truncated 65-amino acid peptide. This observation suggested the existence of RdxA-independent MNZ metabolism mechanisms. Therefore, to explore these mechanisms, we conducted genomic and transcriptomic analyses comparing ICDC15003s with *in vitro*-induced MNZ resistant strains.

## 2 Materials and methods

### 2.1 Strain and culture

The *H. pylori* strain ICDC15003s was isolated in 2015 from a 10-year-old child in Beijing. This strain exhibited significant MNZ susceptibility (MIC = 0.38 μg/mL) (Supplementary Figure S1) and contained a premature termination codon in the *rdxA* gene, resulting in a 65-amino acid truncated RdxA protein (Supplementary Figure S2). The strain was cultured on Karmali blood agar plates (with 5% fresh defibrinated sheep blood) and incubated for 48 h under microaerobic condition (5% O_2_, 10% CO_2_, 85% N_2_) at 37°C.

### 2.2 *In vitro* induction of MNZ resistance

The induction of MNZ resistance was performed according to the method in our previous research with a little modification (Han et al., 2021). Briefly, ICDC15003s was used as initial strain and cultured on Kamarli plates for 48 h, then a suspension of 10^9^ colony forming units per milliliter (CFU/mL) was transferred onto fresh Kamarli plates containing MNZ concentration of 1 μg/mL. The screened single colony was selected and inoculated on the plates with higher concentration of MNZ (8 and 50 μg/mL, respectively). After pressure screening, MNZ-resistant clones (ICDC15003-8 and ICDC15003-50) were obtained. Ten colonies from each of strains ICDC15003-8 and ICDC15003-50 were selected randomly, and the MICs of MNZ were determined using the Etest method (Sun et al., 2024). The stability of MNZ resistance in the screened *H. pylori* isolates, induced by various MNZ concentrations, was ensured by serially subculturing for three generations.

### 2.3 Determination of MIC value for MNZ

The MIC of MNZ was determined using the Etest method. The isolates were suspended in sterile saline after 48 h of growth, and the McFarland turbidity was adjusted to 2.0. The bacterial suspension was spread onto Karmali blood agar plates (with 5% fresh defibrinated sheep blood) and MNZ E-test strips were applied. Plates were incubated for 48 h under microaerobic condition (5% O_2_, 10% CO_2_, 85% N_2_) at 37°C. According to EUCAST guidelines, the strains with MIC >8 μg/mL were considered MNZ resistant.

In addition, MICs of other antibiotics (amoxicillin, clarithromycin, tetracycline, levofloxacin, and gentamicin) were similarly determined for strains ICDC15003s, ICDC15003-8, and ICDC15003-50.

### 2.4 Genomic DNA extraction, genome sequencing, and comparative analysis

Two colonies were selected from each of strains ICDC15003-8 and ICDC15003-50. Genomic DNA was extracted from these four colonies, as well as from the initial strain ICDC15003s, using the QIAamp DNA Mini Kit (Qiagen, Germany) according to the manufacturer’s instructions and quantified by Qubit Fluorometer (Thermo Scientific). Sequencing libraries were generated using the NEBNext Ultra Directional DNA Library Prep Kit for Illumina (NEB, United States) following manufacturer’s recommendations and the constructed libraries were sequenced using PacBio Sequel and Illumina NovaSeq PE150 at the Beijing Novogene.

The raw data were filtered to obtain clean data and specific processing steps were performed to assemble genome using the clean data. Genome annotation was performed using Prokka and Snippy was employed to analyze the SNP (Single Nucleotide Polymorphism), finally SnpEFF was used for SNP annotation, to complete the comparative genomic analysis.

Furthermore, the sequences of *rdxA*, *frxA*, and *fdxB* genes were extracted from genomes of strains ICDC15003s, ICDC15003-8, and ICDC15003-50. The amino acid sequences of these genes were compared with those from eight MNZ-susceptible strains, as well as *H. pylori* 26695 using alignment software. Additionally, the PCR of *rdxA* genes of strains ICDC15003s, ICDC15003-8, and ICDC15003-50 was carried out. The primer set was F-GCAGGAGCATCAGATAGTTCT and R-GGGATTTTATTGTATGCTACAA, which would yield a product size of 886 bp, including the *rdxA* gene of 630 bp. The PCR products were sequenced by the Sangon Company (Shanghai, China) and the sequences were compared using alignment software.

### 2.5 RNA extraction and library construction

Transcriptome analysis was performed on strain ICDC15003s, ICDC15003-8 and ICDC15003-50. After 48 h of culture, the *H. pylori* isolates were harvested, and total RNA was extracted using the E.Z.N.A. Bacteria RNA kit (Omega Bio, United States) according to manufacturer protocols. Three replicates were performed for each strain.

The quality control of RNA was performed using RNA Nano 6000 Assay Kit of the Bioanalyzer 2100 system (Agilent Technologies, CA, United States) to ensure the RNA integrity. Subsequently, cDNA libraries were constructed using NEBNext Ultra Directional RNA Library Prep Kit (NEB, United States).

### 2.6 RNA sequencing

The constructed libraries were sequenced using Illumina Hiseq platform, and the libraries construction and sequencing were carried out in Beijing Novogene Bioinformatics Technology Co., Ltd. Clean reads were obtained by removing reads containing adapters, bases of low quality and mapped to the ICDC15003 genome using Bowtie2 (Langmead, B. 2012). The mapped reads were quantified for each gene using HTSeq v0.6.1.

### 2.7 Differential expression, GO, and KEGG pathway analysis

Differentially expressed genes between strain ICDC15003s and strain ICDC15003-50, including biological replicates, were analyzed using the DESeq R package (1.18.0). Genes with a *p-*value <0.05 and an absolute log2 (fold change) >1 adjusted for multiple testing using the Benjamini–Hochberg method, were considered differentially expressed. Differentially expressed genes were classified using the Clusters of Orthologous Groups of Proteins (COGs) Database.

Gene Ontology (GO) and Kyoto Encyclopedia of Genes and Genome (KEGG) enrichment were performed to analyze the functions of differentially expressed genes using clusterProfiler R package (3.8.1) (Yu et al., 2012). A significance threshold of *p* < 0.05, adjusted for multiple testing, was applied for both GO and KEGG pathway enrichment analyses.

### 2.8 Quantitative real-time PCR

The cDNA synthesis and quantitative real-time PCR (qRT-PCR) were performed using the one-step qRT-PCR superMix kit (Transgen Biotech, Beijing, China). Four genes identified as differentially expressed through RNA sequencing were selected for validation. Data collection was conducted using the ABI QuantStudio6 system according to the manufacturer’s instructions. *H. pylori* 16S rRNA was used as an internal control. The primers and probes used for these genes were shown in Table 1. Relative fold changes in gene expression were determined using the threshold cycle ( $2^{-\mathrm{\Delta \Delta CT}}$ ) method. Three replicates for each gene were performed.

**Table 1:** Quantitative real-time PCR primers used in this study.

Gene ID	Primers	Probes
697610AGL000416	F-TTCGCTCCAAAACATCAATATTTC	CGCCCACGATCGCCACCCT
	R-GAGCTTTTCCCGCTCCCG	
697610AGL000530	F-AAGTGGCTCTTTTAAAAGGGGC	AGGTGCTTTGCGTGGATGTGGGG
	R-TTTCTGGCGTTTTAAACCCTCT	
697610AGL000797	F-AGCAAGCAGACTTATATCCAAATG	GCGAACATGATGATGGCAGCGG
	R-CCTTCAATCGGGCAAGAATC	
697610AGL001031	F-AGGACTAACCCTGATGTGAATGTG	GGAGGGAGCGTGATGGGGCA
	R-CACGCTTTTGAGCATGCCA	
16S rRNA	F-CGGAATCACTGGGCGTAAAG	GCGTAGGCGGGATAGTCAGTCAGGTG
	R-CCACCTACCTCTCCCACACTCT	

The probes were fluorescently labeled with FAM in 5′ and BHQ-1 in 3′ position. 697610AGL000416, ABC transporter ATP-binding protein (yejF); 697610AGL000530, TlyA family RNA methyltransferase; 697610AGL000797, NADPH-flavin oxidoreductase; 697610AGL001031, TonB-dependent receptor.

### 2.9 Data visualization

A variety of figures were generated using the R 4.2.2 language and environment. The design of volcano map and heatmap was accomplished using the R packages ggplot2, ComplexHeatmap and RColorBrewer.

## 3 Results

### 3.1 *In vitro* induction of MNZ-resistant isolates from strain ICDC15003s

After pressure induction with continuous MNZ concentration, the initial MNZ-susceptible strain ICDC15003s successfully developed resistance at MNZ concentrations of 8 μg/mL (ICDC15003-8) and 50 μg/mL (ICDC15003-50), respectively. All selected isolates exhibited high-level resistance (MIC >256 μg/mL), regardless of the induced MNZ concentration. Additionally, the resistance profiles of ICDC15003s, ICDC15003-8 and ICDC15003-50 were consistent for other antibiotics, with MIC values of 0.016 μg/mL for amoxicillin, 0.19 μg/mL for clarithromycin, 1.5 μg/mL for tetracycline, 0.75 μg/mL for levofloxacin and 0.5 μg/mL for gentamicin. These results indicated that the induction process did not affect the resistance of these strains to other antibiotics.

### 3.2 Genome alignment of ICDC15003s, ICDC15003-8, and ICDC15003-50

Genome sequences of each two colonies of ICDC15003-8 (ICDC15003-8-1 and ICDC15003-8-2) and ICDC15003-50 (ICDC15003-50-1 and ICDC15003-50-2) were compared with that of initial ICDC15003s. We found that several gene mutations were present and varied between different colonies. SNP annotation revealed missense mutations in genes including *yfkO*, *acxB*, *alr1*, *glk*, *cobB*, and *rpsC* (Table 2 and Supplementary Figures S3–S8).

**Table 2:** The genes with missense mutations by comparative genomic analysis.

Gene	Mutation			
	ICDC15003-8-1	ICDC15003-8-2	ICDC15003-50-1	ICDC15003-50-2
Putative NAD(P)H nitroreductase (*yfkO*)	C → T	—	—	—
Acetone carboxylase alpha subunit (*acxB*)	G → A	—	—	—
Alanine racemase 1 (*alr*1)	—	—	—	C → T
Glucokinase (*glk*)	—	—	T → C	—
Hypothetical protein	—	—	—	T → G
30S ribosomal protein S3 (*rpsC*)	—	—	G → A	—
NAD-dependent protein deacylase (*cobB*)	C → T	—	—	—

ICDC15003-8-1 and ICDC15003-8-2 indicated two colonies of ICDC15003-8 which were screened with MNZ concentration of 8 μg/mL; ICDC15003-50-1 and ICDC15003-50-2 indicated two colonies of ICDC15003-50 which were screened with MNZ concentration of 50 μg/mL. The genes with mutations were compared with those from ICDC15003s.

**Figure 1:**
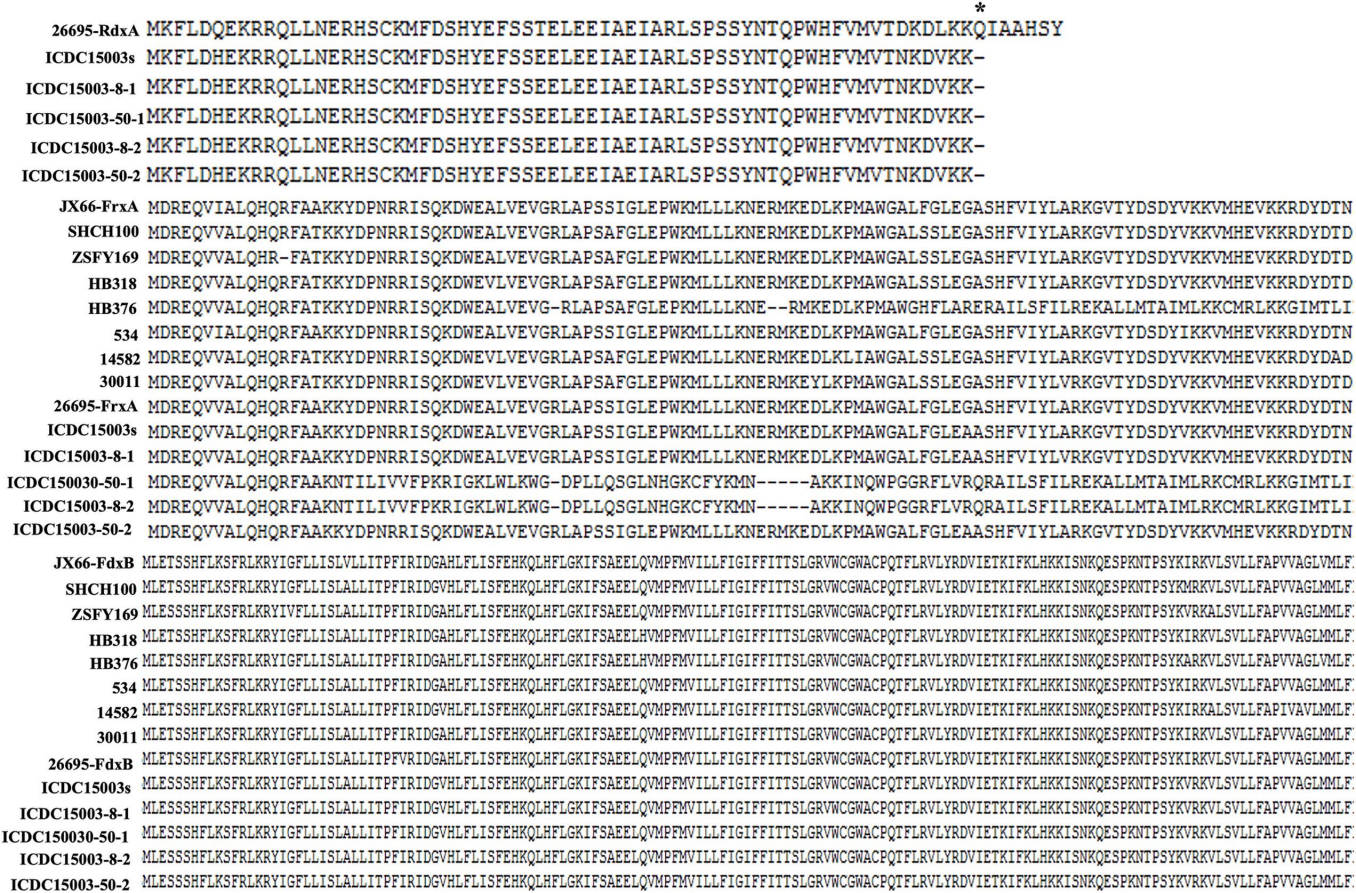
The sequence alignment of RdxA, FrxA, and FdxB between MNZ-susceptible strains and ICDC15003. Strains JX66, SHCH100, ZSFY169, HB318, HB376, 534, 14582, and 30011 were MNZ susceptible.

We further analyzed key genes related to MNZ metabolism, including *rdxA*, *frxA*, and *fdxB* to determine if the induction process led to changes in these genes. Alignment results showed that the nonsense mutation in *rdxA* was consistent between the initial and resistant strains, as confirmed by both genome sequencing and PCR product sequencing (Supplementary Figures S9–S12). In addition, the amino acid sequences of FdxB remained unchanged. However, compared to *H. pylori* 26695, several mutations were observed in our strains, such as T8S, V37I, and A42V, which were also present in other susceptible strains. Additionally, several amino acid substitutions were present in FrxA in our strains, including G76A, I118F, and N124D. Notably, frameshift mutations at amino acid position 18 were observed in strain ICDC15003-8-2 and ICDC15003-50-1 (Figure 1).

### 3.3 Transcriptome analysis on strain ICDC15003s and ICDC15003-50-1

#### 3.3.1 Identification of differentially expressed genes

**Figure 2:**
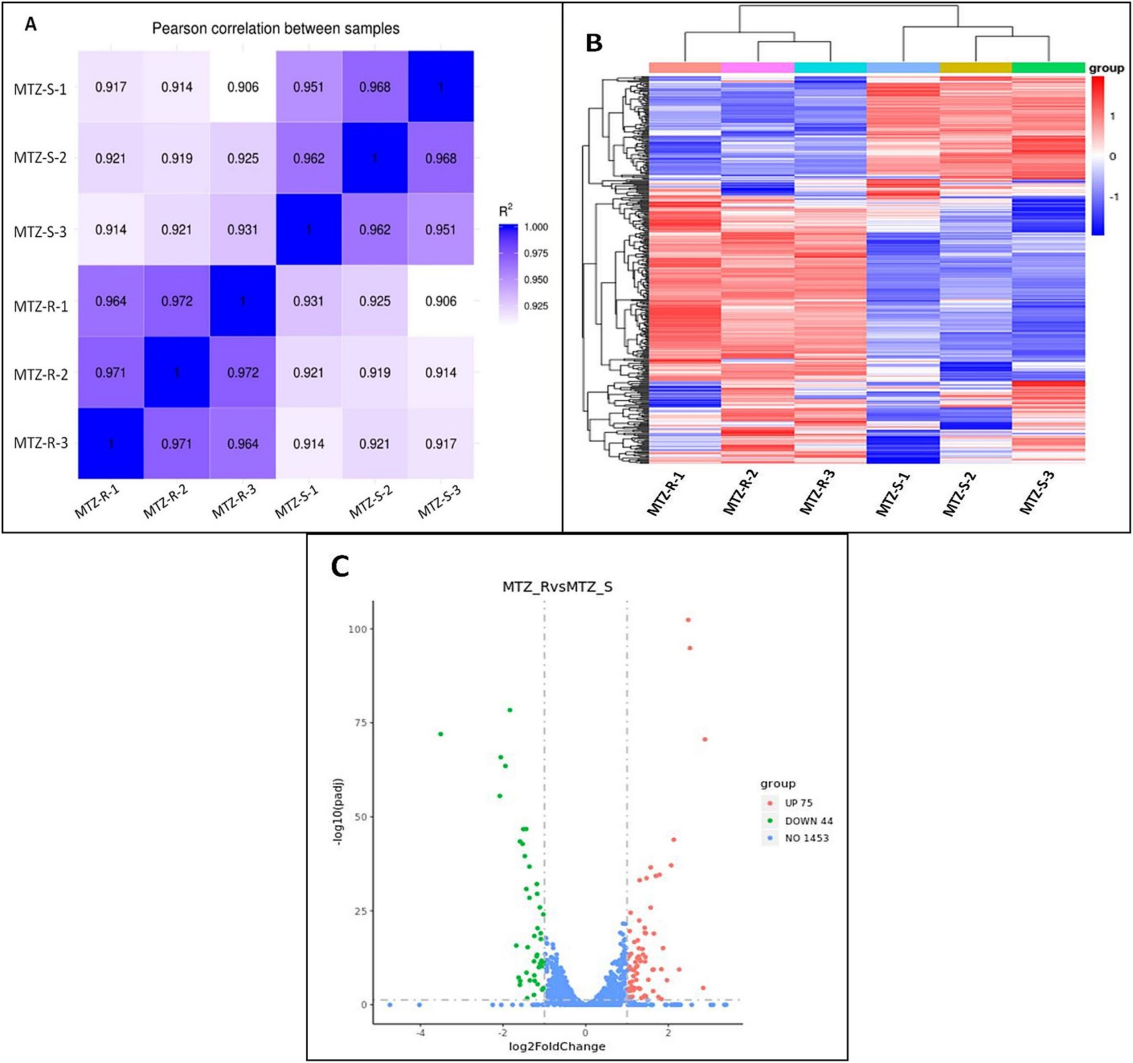
Differentially expressed genes in strain ICDC15003s (MNZ-S) and ICDC15003-50-1 (MNZ-R). **(A)** Pearson correlation heat map of all samples. The values indicated the Pearson correlation coefficient square (*R*^2^). **(B)** The clustering heat map of differential expression. MNZ-R indicated three replicates of strain ICDC15003-50-1, and MNZ-S indicated three replicates of strains ICDC15003s. The red color corresponded to up-regulated genes and blue indicated down-regulated genes. **(C)** The volcano map of differential analysis. The horizontal axis indicated the log2 fold change, and vertical axis indicated the −log10(*p*adj). The up-regulated, down-regulated, and unchanged genes in ICDC15003-50-1 compared with those of ICDC15003s were represented as red, green, and blue dots, respectively. The numbers indicate the total number of genes.

Transcriptome analysis of strains ICDC15003s and ICDC15003-50-1 revealed consistent gene expression patterns across three replicates of both strains (Figure 2A). A total of 119 genes were differentially expressed (log2 fold change >1 or <−1) in strain ICDC15003-50-1, including 75 up-regulated genes and 44 down-regulated genes (Supplementary Table S1 and Figures 2B,C).

#### 3.3.2 GO and KEGG pathway enrichment analysis

GO and KEGG pathway enrichment analyses were performed to assess the functions of differentially expressed genes. Significant enrichment of up-regulated genes was observed for peptide, amide, and protein metabolic process, as well as cellular macromolecule biosynthetic process. Cellular components enriched included ribosome, intracellular organelle, cytoplasmic part, non-membrane-bounded organelle, and cell part. Molecular function enrichment was observed for structural constituent of ribosome and molecule activity (Supplementary Table S5). Additionally, only ribosome pathway showed significant enrichment in the KEGG analysis. A subset of genes enriched in GO analysis were list in Table 3.

**Table 3:** Part genes enriched in GO analysis for strain ICDC15003-50-1.

Gene ID	Log2 (FC)	Description	*p*adj
697610AGL000953	1.430	Molybdenum cofactor biosynthesis protein (MoaE)	0.003
697610AGL000150	1.430	Pyridoxine 5′-phosphate synthase (PdxJ)	7.15 × 10^−20^
697610AGL001294	1.241	Glucose-6-phosphate isomerase	4.51 × 10^−9^
697610AGL000907	1.409	Flagellin-specific chaperone (FliS)	0.006
697610AGL000069	1.076	ABC transporter permease (Mla)	0.008
697610AGL000640	1.373	Neuraminyllactose-binding hemagglutinin (HpaA)	1.47 × 10^−15^

### 3.4 Transcriptome analysis on strain ICDC15003s and ICDC15003-50-2

#### 3.4.1 Identification of differentially expressed genes

**Figure 3:**
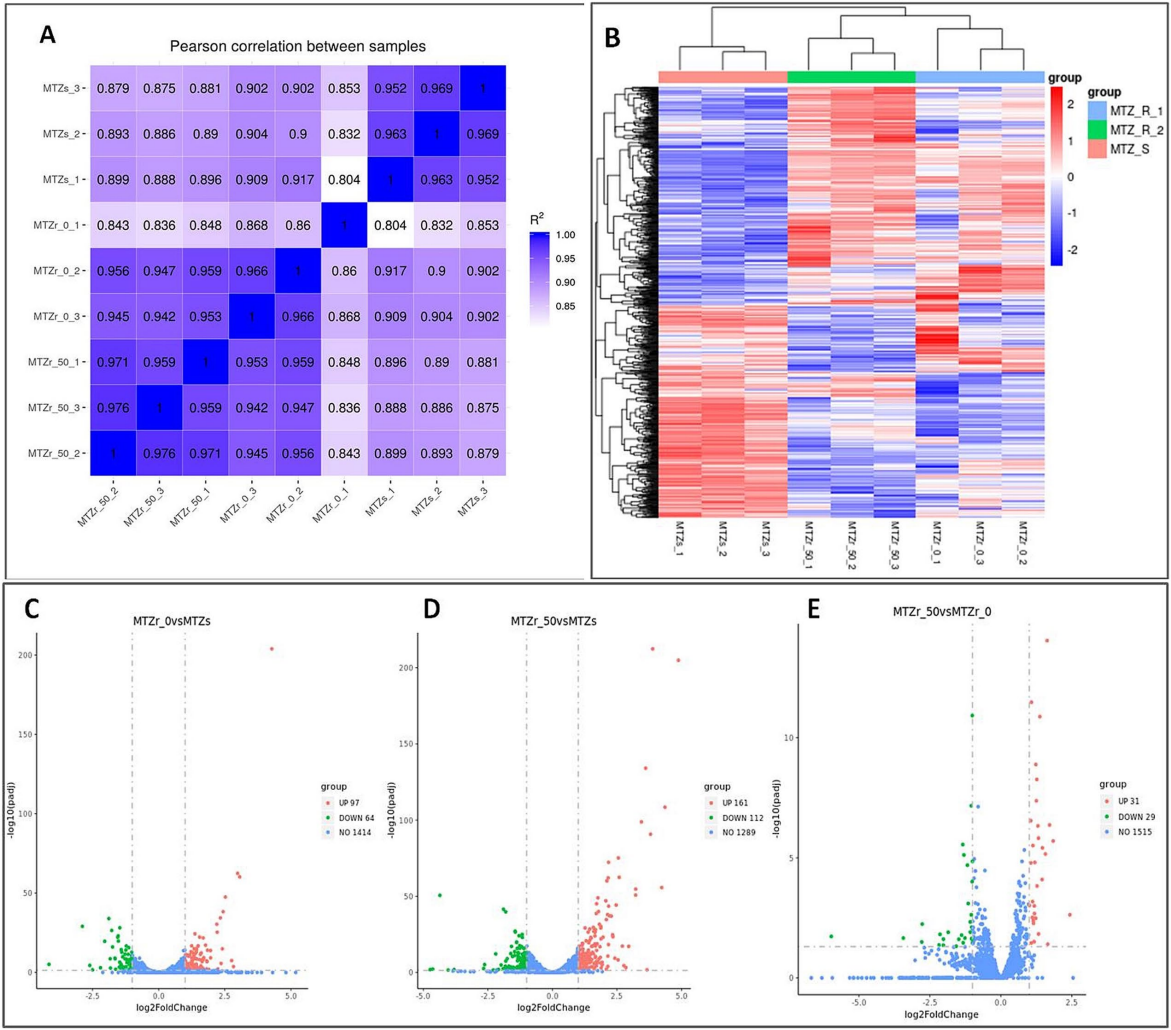
Differentially expressed genes in strain ICDC15003s (MNZ-S) and ICDC15003-50-2 (MNZ-R). MNZ r0 and MNZ r50 indicated the ICDC15003-50-2 strain cultured on plates without or with MNZ, respectively. **(A)** Pearson correlation heat map of all samples. The values indicated the Pearson correlation coefficient square (*R*^2^). **(B)** The clustering heat map of differential expression. MNZ-R indicated three replicates of strain ICDC15003-50-2, and MNZ-S indicated three replicates of strains ICDC15003s. The red color corresponded to up-regulated genes and blue indicated down-regulated genes. **(C–E)** The volcano map of differential analysis. The horizontal axis indicated the log2 fold change, and vertical axis indicated the −log10(*p*adj). The up-regulated, down-regulated, and unchanged genes in ICDC15003-50-2 compared with those of ICDC15003s were represented as red, green, and blue dots, respectively. The numbers indicate the total number of genes.

Strain ICDC15003-50-2 was cultured on plates without MNZ (MNZ r0) and with the MNZ concentration of 50 μg/mL (MNZ r50), respectively. Transcriptome analysis of strains ICDC15003s, MNZ r0, and MNZ r50 showed consistent gene expression patterns across three replicates of each group (Figure 3A). In MNZ r0, 161 genes were differentially expressed (log2 fold change >1 or <−1), including 97 up-regulated and 64 down-regulated genes (Supplementary Table S2 and Figures 3B,C). In MNZ r50, 273 genes were differentially expressed, including 161 up-regulated and 112 down-regulated genes (Supplementary Table S3 and Figure 3D). Additionally, 60 genes were differentially expressed between MNZ r50 and MNZ r0, including 31 up-regulated and 29 down-regulated genes (Supplementary Table S4 and Figure 3E).

#### 3.4.2 GO and KEGG pathway enrichment analysis

In the MNZ r0 group, significant enrichment was observed for up-regulated genes involved in cofactor metabolic process and cofactor biosynthetic process, such as *pdxJ*, *pdxA* and *nifU* (Supplementary Table S6 and Table 4).

**Table 4:** MNZ r0 vs. MNZ s up GO enrich significant.

Gene ID	Log2 (FC)	Gene	*p*adj
697610AGL000150	2.984	Pyridoxine 5′-phosphate synthase (*pdxJ*)	3.15 × 10^−63^
697610AGL000151	2.522	4-hydroxythreonine-4-phosphate dehydrogenase (*pdxA*)	3.02 × 10^−48^
697610AGL001478	1.226	Phosphoglycerate kinase	1.04 × 10^−13^
697610AGL001411	1.320	Thiamine diphosphokinase	3.56 × 10^−9^
697610AGL000558	1.254	3-methyl-2-oxobutanoate hydroxymethyltransferase	5.70 × 10^−8^
697610AGL000386	1.028	Iron-sulfur cluster assembly scaffold protein NifU	3.29 × 10^−7^
697610AGL000320	1.053	Phosphopyruvate hydratase	6.99 × 10^−6^

When comparing MNZ r50 and MNZ r0 groups, significant enrichment of up-regulated genes was observed for peptide, amide and protein metabolic process, as well as cellular macromolecule biosynthetic process. Cellular component enriched included ribosome, intracellular organelle, cytoplasmic part, non-membrane-bounded organelle, and cell part. Molecular function enrichment was observed for structural constituent of ribosome and molecule activity (Supplementary Table S7).

In the KEGG analysis, up-regulated genes in the arginine and proline metabolism pathway were significantly enriched in the MNZ r0 group, including aliphatic amidase, *aspB*, *speE* and arginase (Table 5). Additionally, genes involved in oxidative phosphorylation and ABC transporters pathway were significantly down-expressed, such as NADH-quinone oxidoreductase, *metQ*, *dppB*, and *dppD* (Table 5).

**Table 5:** MNZ r0 vs. MNZ s KEGG rich significant.

Gene ID	Log2 (FC)	Gene	*p*adj
697610AGL000460	2.198	Aliphatic amidase	3.99 × 10^−31^
697610AGL000826	1.490	Aspartate aminotransferase (*aspB*)	2.14 × 10^−17^
697610AGL000991	1.421	Spermidine synthase (*speE*)	1.66 × 10^−13^
697610AGL000089	1.125	Arginase	2.53 × 10^−11^
697610AGL001404	−1.072	NADH-quinone oxidoreductase subunit (*nuoM*)	4.86 × 10^−9^
697610AGL001401	−1.101	NADH-quinone oxidoreductase subunit (*nuoJ*)	7.78 × 10^−8^
697610AGL001399	−1.009	NADH-quinone oxidoreductase subunit (*nuoH*)	1.96 × 10^−7^
697610AGL001402	−1.061	NADH-quinone oxidoreductase subunit (*nuoK*)	0.000515
697610AGL000132	−1.773	ABC transporter substrate-binding protein (*metQ*)	3.24 × 10^−27^
697610AGL000465	−1.273	ABC transporter permease (*dppB*)	3.20 × 10^−12^
697610AGL000466	−1.020	ABC transporter permease (*dppC*)	1.86 × 10^−11^
697610AGL000417	−1.082	ABC-type microcin C transport system, permease component (*yejE*)	3.59 × 10^−8^
697610AGL000467	−1.136	ABC transporter ATP-binding protein (*dppD*)	4.46 × 10^−8^
697610AGL000416	−2.498	ABC-type microcin C transport system, duplicated ATPase component (*yejF*)	0.02156533

#### 3.4.3 The differentially expressed genes in three MNZ groups

**Figure 4:**
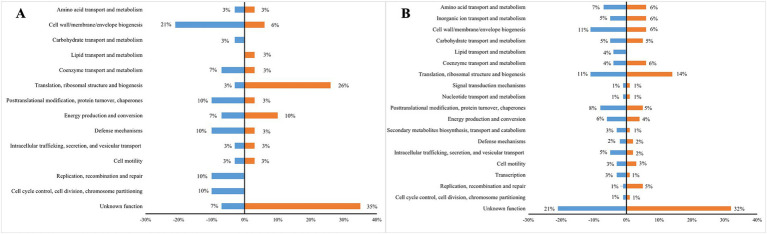
The COGs functional annotation of the differentially expressed genes in three MNZ strains compared to ICDC15003s. **(A)** Differentially expressed genes between ICDC15003-MNZ r50 and MNZ r0. The blue indicates down-regulated genes, and the orange indicates up-regulated genes. **(B)** Differentially expressed genes in ICDC15003-50-1 and MNZ r0 compared to ICDC15003s. The blue indicates down-regulated genes, and the orange indicates up-regulated genes. The abundance of each category is represented as a percentage.

COGs functional annotation showed that the gene expression patterns between MNZ r50 and MNZ r0 were significantly different. Among down-regulated genes, those involved in cell wall/membrane/envelope biogenesis accounted for a larger proportion (21%), while genes involved in translation, ribosomal structure and biogenesis accounted for 26% of up-regulated genes. Categories including “replication, recombination and repair,” “carbohydrate transport and metabolism” as well as “cell cycle control, cell division, chromosome partitioning” were observed in down-regulated genes, while genes belonging to the “lipid transport and metabolism” category were up-regulated (Figure 4A).

In MNZ-resistant strains, the predominant functional category was translation, ribosomal structure, and biogenesis, including genes encoding translation elongation factor EF-G, methionine aminopeptidase, threonylcarbamoyltransferase TsaD, and several Ribosomal proteins. Conversely, the expression of genes involved in lipid transport and metabolism, decreased in MNZ-resistant strains, including 3-hydroxyisobutyrate dehydrogenase, acetyl-CoA synthetase and CDP-diacylglycerol pyrophosphatase (Figure 4B).

Moreover, 23 up-regulated genes were observed in all three groups, such as *pdxj*, *mod*, and *fecA* (Table 6).

**Table 6:** Part up-regulated expressed genes in three MNZ strains.

Gene ID	Gene description	COG classification
697610AGL000007	Site-specific DNA-methyltransferase (*mod*)	Replication, recombination, and repair
697610AGL000150	Pyridoxine 5′-phosphate synthase (*pdxJ*)	Coenzyme transport and metabolism
697610AGL000386	Iron-sulfur cluster assembly scaffold protein (*nifU*)	Posttranslational modification, protein turnover, chaperones
697610AGL000460	Aliphatic amidase	Amino acid transport and metabolism
697610AGL000838	TonB-dependent receptor family protein (*fecA*)	Inorganic ion transport and metabolism
697610AGL000907	Flagellar export chaperone (*fliS*)	Cell motility
697610AGL001031	TonB-dependent receptor	Inorganic ion transport and metabolism
697610AGL001233	Pyruvate flavodoxin oxidoreductase subunit gamma (*porG*)	Energy production and conversion
697610AGL001353	Rhodanese-like domain-containing protein	Inorganic ion transport and metabolism
697610AGL001201	Thioredoxin fold domain-containing protein (*dsbC*)	Posttranslational modification, protein turnover, chaperones

### 3.5 qRT-PCR verifying the gene expression

**Figure 5:**
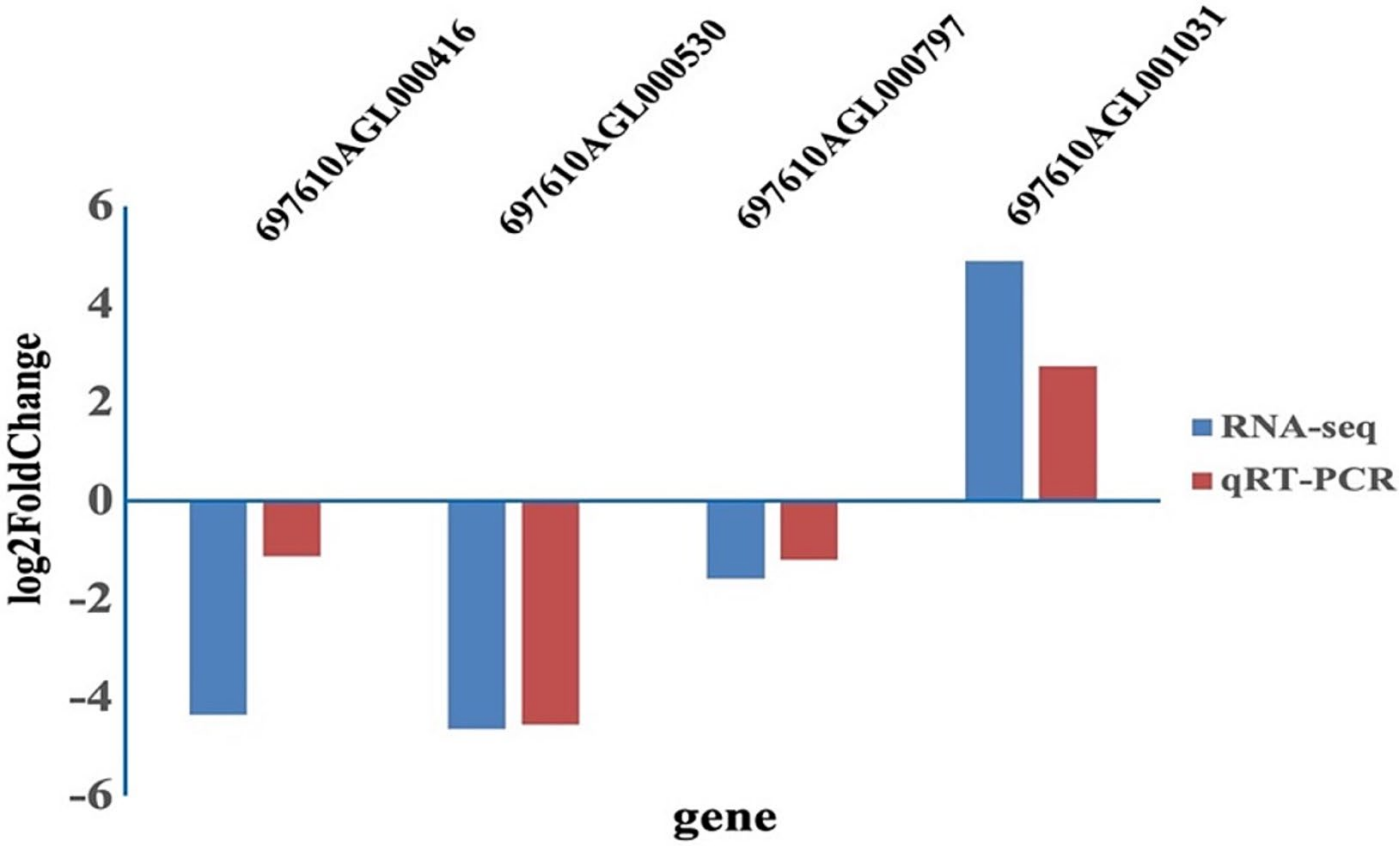
The log2 fold change for four differently expressed genes using qRT-PCR analysis and RNA-seq.

As shown in Figure 5, the expression levels were consistent between RNA-seq and qRT-PCR. The genes encoding *yejF*, *tlyA* and oxidoreductase were down-regulated in resistant strains, while the gene encoding TonB-dependent receptor was up-regulated.

## 4 Discussion

In this study, we successfully induced high-level MNZ resistance in the *H. pylori* strain ICDC15003s *in vitro* and conducted genomic and transcriptomic profiling. We found genetic mutations or expression changes in several genes, particularly, we proposed that NADH-quinone oxidoreductase might serve as an RdxA-independent mechanism for MNZ metabolism and resistance. This is the first study to explore mechanisms that expand the metabolism and resistance mechanisms of RdxA in *H. pylori*.

We identified missense mutations in several genes such as *acxB*, *alr1*, *yfkO*, *glk*, and *cobB* in resistant strains. AcxB, a subunit of acetone carboxylase in *H. pylori*, may function in acetone utilization or catalyze a related reaction that is important for growth in the host (Brahmachary et al., 2008). Mutation in *alr1*, which encoded the alanine racemase, was observed in strain ICDC15003-50-2, and its expression level decreased in MNZ r50 in the presence of MNZ (Supplementary Table S4). This suggested that the mutation might be involved in gene expression regulation under stress. YfkO nitroreductase from *Bacillus licheniformis* is capable of reducing nitro-compounds to the corresponding hydroxylamine derivatives (Ball et al., 2021). However, there is no study on the specific function of YfkO protein in *Helicobacter pylori*.

After MNZ resistance induction, multiple genes exhibited altered expression. GO or KEGG pathways analyses revealed enrichment in cofactor metabolic and biosynthetic process, arginine and proline metabolism pathway, and ABC transporters pathway. Notably, the most significantly up-regulated genes included *pdxJ* and *pdxA*, which are involved in vitamin B6 synthesis and are necessary for the synthesis of glycosylated flagella and flagellum-based motility of *H. pylori* (Grubman et al., 2010). The ATP-binding cassette (ABC) transporters, including *dppB*, *dppC*, and *dppD*, which are responsible for dipeptides and some oligopeptides transportation (Xu et al., 2021), showed reduced expression levels in resistant strains. The down-regulation of dipeptides transporter significantly reduced the peptides use ability and caused deficiencies in bacterial growth (Rahman et al., 2019; Weinberg and Maier, 2007). Additionally, multiple mutations in *dppB* were found in MNZ-resistant strains (Miftahussurur et al., 2017). Our previous study demonstrated that these genes were associated with amoxicillin resistance, via altering the structure and permeability of outer membrane (Han et al., 2021). The genomic and transcriptomic results suggested that these genes were not directly involved in MNZ metabolism but were probably related to drug resistance.

RdxA, an oxygen insensitive NADH (NADPH) nitro reductase encoded by *rdxA* gene, is the primary enzyme for MNZ activation in *H. pylori*. RdxA directly transfers two electrons to MNZ using NADH (NADPH) and FMN, forming toxic nitroso derivatives (Olekhnovich et al., 2009). Previous studies showed that MNZ sensitivity could be restored in *H. pylori*-resistant strains by introducing functional RdxA with shuttle plasmid (Goodwin et al., 1998). In addition to RdxA, other components of electron transport chain, including NAD(P)H flavin oxidoreductase FrxA, ferredoxin FdxA, FdxB, FldA, 2-ketoglutarate oxydoxin reductase (OorD), pyruvate ferredoxin (PorD), may also be related to MNZ metabolism (Chua et al., 2019; Smith and Edwards, 1997). The most studied component is FrxA, and previous study demonstrated that high expression of FrxA could counteract the effect of RdxA mutation and render MNZ susceptible (Chua et al., 2019). However, the amino acid 16 mutation in RdxA generally results in moderate MNZ resistance. The study by Losurdo G (Losurdo et al., 2022) indicated that MNZ resistance could not affect the eradication effect based on determination of *rdxA* and *frxA* mutations, suggesting that other mechanisms were likely involved in MNZ metabolism and resistance. Previous study has demonstrated that in *rdxA* alleles that show mutations leading to stop codons, which results in truncated polypeptides, the corresponding RdxA will not be functional (Mendz and Mégraud, 2002). Moreover, our previous study revealed that truncations occurring before the 70th amino acid position are associated with higher level of resistance. This is likely because a truncated RdxA fragment shorter than 70 amino acids significantly impairs both FMN cofactor binding affinity and dimer formation, which are essential for its metabolic activity, leading to a complete loss of the RdxA function and subsequent rapid degradation (Gong et al., 2023). *H. pylori* exhibits distinct population structures across geographical regions, with substantial genetic diversity observed in *rdxA* gene among different strains (Zhang et al., 2020). In our study, alignment of *rdxA* sequences between clinical strain ICDC15003s and the reference European strain *H. pylori* 26695 revealed multiple mutations (Supplementary Figures S10–S12), which likely reflects the genetic differences between Western and East Asian strains. These sites may be indicative of the phylogenetic characteristics of *H. pylori* strains originating from China. Moreover, we found the strain ICDC15003s contained a premature termination codon in the *rdxA* gene, resulting in a 65-amino acid truncated RdxA protein (Supplementary Figure S2). This shorter truncation is expected to completely abolish RdxA activity and promote its rapid degradation. Additionally, the sequences, and gene expression levels of the reported nitro reductases, including FrxA, FdxB, FdxA, OorDABC, and PorCDAB, remained unchanged after MNZ resistance induction, indicating that these components were not responsible for MNZ sensitivity in ICDC15003s.

Beyond proteins with nitroreductase activity that directly catalyze MNZ, the cellular machinery regulating redox status and maintaining low intracellular potential is critical to indirectly activate MNZ. Previous studies showed that the redox enzyme activity of resistant strains was significantly lower than that of sensitive strains (Kaakoush and Mendz, 2005). The key difference between MNZ-sensitive and resistant strains was that the former could maintain the hypoxic state of the MNZ-reducing site by enhancing NADH oxidase activity (Smith and Edwards, 1997). In our study, compared with strain ICDC15003s, changes in the transcription of genes encoding NADH-quinone oxidoreductase subunit M, J, H, and K were detected in MNZ r0, with down-regulated expression. NADH-quinone oxidoreductase was a muti-subunit protein complex and the first protein of electron transport chain (Lee et al., 2013). The complex protein catalyzes the first step of oxidative phosphorylation by transferring two electrons from NADH to ubiquinone, coupling the redox reaction to proton translocation and thus conserving the redox energy in a proton gradient (Mugengana et al., 2021). As a core component of respiratory chain, NADH-quinone oxidoreductase is highly conserved in structure and function across all aerobic and microaerobic organisms. In *H. pylori*, it plays a crucial role in energy metabolism and redox balance. Elevated expression of this complex may enhance the intracellular redox capacity, promoting the conversion of NADH into NAD+, thereby providing a more abundant electron donor for the reductive activation of MNZ. Additionally, previous study has suggested that NADH-quinone oxidoreductase may be involved in benzimidazole resistance, by altering the drug into active anti-*H. pylori* agents, similar to the action of RdxA (Mills et al., 2004). This highlights that changes in gene expression can influence the cellular redox potential and the concentration of bactericidal intermediates, thereby contributing to resistance. Therefore, based on transcriptome analyses, several genes that affected intracellular redox potential, such as some components of the NADH-quinone redox reductase complex, may potentially compensate for the loss of RdxA function.

**Figure 6:**
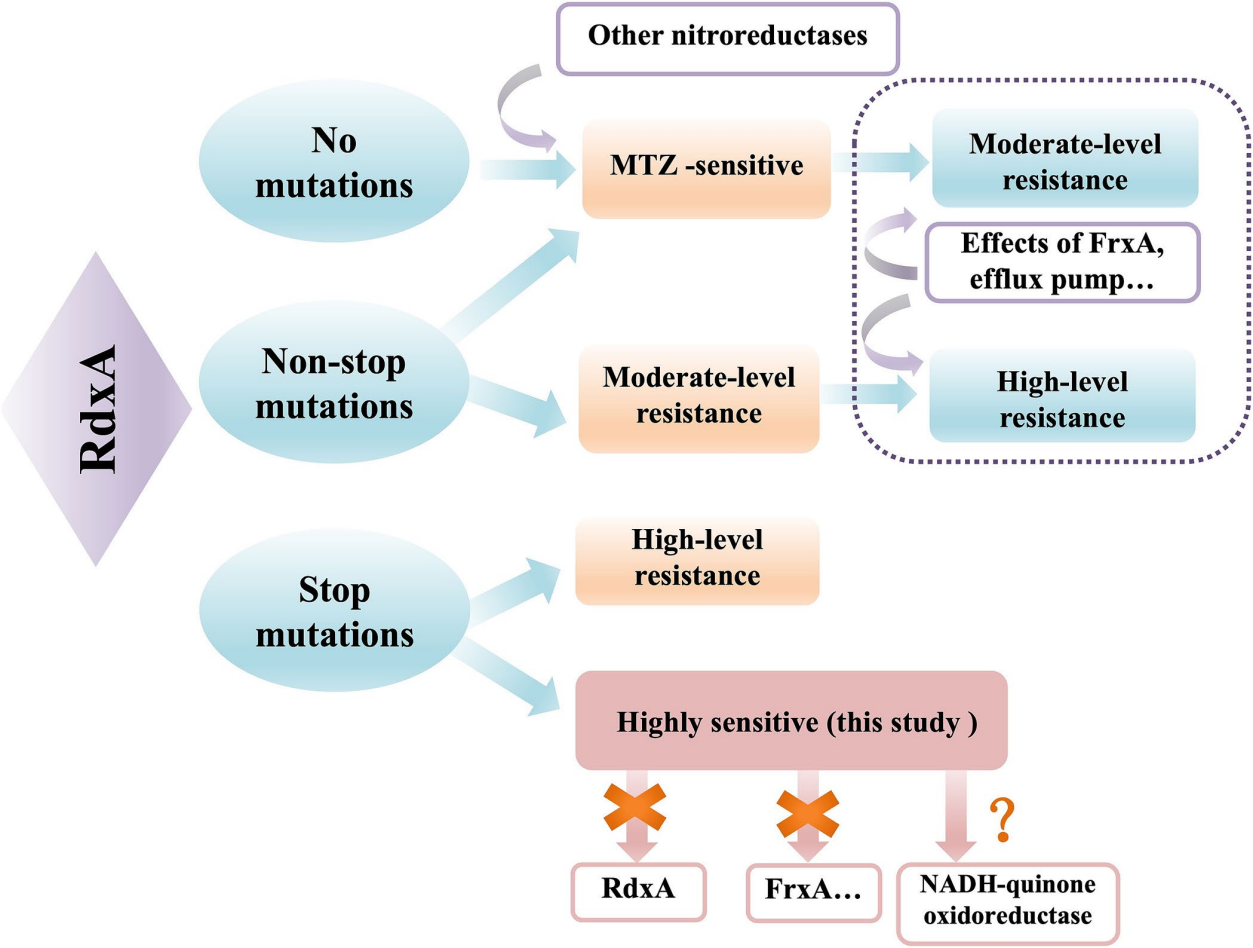
The relationship between RdxA mutation and MNZ metabolism. RdxA is the main mechanism of MNZ sensitivity in *H. pylori*, while other catalytic enzymes play a relatively small role. Under the effects of FrxA mutation or efflux pump, the level of resistance caused by RdxA non-termination mutation can be increased or the level of resistance of sensitive strains can be induced. RdxA termination mutations directly lead to high-level drug resistance, and the highly sensitive strain in this study adopted other mechanisms independent of RdxA.

In our study, we summarized the relationship between RdxA function and MNZ metabolism, as well as resistance (Figure 6). RdxA is the determinant of MNZ metabolism and resistance in *H. pylori*, and the type of mutation is related to the level of MNZ resistance. Mutations in other components, such as FrxA or efflux pump, may potentially increase the level of resistance caused by RdxA mutations (Albert et al., 2005). Cumulative effects probably occurred in combination with RdxA truncated mutations, which could account for the high-level resistance observed after induction in our study. RdxA termination mutations directly lead to high level of MNZ resistance, while an RdxA-independent mechanism of MNZ metabolism was first reported in this study. There were several limitations in this study. Firstly, the activity of RdxA relies on specific cofactors (such as FMN, NADPH) and reaction conditions (such as hypoxic environments) that are difficult to fully simulate *in vitro*. Moreover, active intermediates produced by RdxA-mediated reduction of MNZ are highly unstable and difficult to quantify. Therefore, we did not experimentally verify the complete loss of RdxA activity in these strains. Further study is necessary to measure RdxA activity by more sensitive detection methods. Secondly, while NADH-quinone oxidoreductase was proposed as an RdxA-independent metabolic mechanism through transcriptomic analyses, this hypothesis has not been tested experimentally. Further studies are necessary to investigate the MNZ metabolic activity of ICDC15003s strain using mass spectrometry and mutations responsible for MNZ resistance using allelic replacement. Thirdly, these findings were primarily derived from laboratory-induced drug-resistant strains, and the sample size was limited. The generalizability and clinical relevance have not yet been validated.

## 5 Conclusion

We identified several genes affecting intracellular reductive potential and proposed that NADH-quinone oxidoreductase might serve as an RdxA-independent metabolic mechanism in *H. pylori*, conferring resistance to MNZ through expression changes. This study expanded our understanding of the mechanisms of MNZ metabolism and resistance in *H. pylori*, particularly highlighting the potential compensatory role of NADH-quinone oxidoreductase in the absence of RdxA activity. These findings could provide a solution to the serious problem of MNZ resistance in *H. pylori* and offer new strategies for the development of nitroimidazole antibiotics.

## Data Availability

The sequences of strains in this study were deposited in the NCBI database (http://www.ncbi.nlm.nih.gov/). The accession numbers for the sequences of ICDC15003-8-1, ICDC15003-8-2, ICDC15003-50-1, ICDC15003-50-2, ICDC15003s were CP134396, CP134395, CP134394, CP134393, and CP134397. The unusual strain ICDC15003s is available to other researchers for non commercial research purposes upon request. Interested parties should contact the corresponding author for further details.
